# Light-RepViTSR: ultra-lightweight super-resolution for real-time photoacoustic endoscopy in tumor biopsy

**DOI:** 10.3389/fbioe.2026.1762967

**Published:** 2026-03-19

**Authors:** Guanyi Jiang, Rui Yang, Zhanfeng Fang, Yuwei Luo, Xianghu Yu, Li Liu

**Affiliations:** 1 Department of Hematology, Peking University Shenzhen Hospital, Shenzhen, China; 2 Dongguan Key Laboratory for Cross-Scale Autonomous Interventional Surgical Robotics, School of Advanced Engineering, Great Bay University, Dongguan, China

**Keywords:** deep-learning, *in situ* biopsy, lightweight neural network, photoacoustic endoscopy, real-time imaging, RepVit, super-resolution

## Abstract

Real-time *in situ* biopsy marks a paradigm shift in clinical oncology by enabling immediate intraprocedural pathological diagnosis during endoscopy. Photoacoustic endoscopy (PAE) is a pivotal technology in this field that uniquely visualizes tumor microvasculature and hypoxia through the synergistic fusion of optical contrast and ultrasonic resolution. However, PAE’s inherent resolution-speed tradeoff in raster scanning induces severe motion artifacts from physiological activities (e.g., peristalsis and respiration), critically compromising diagnostic reliability. Although deep-learning-based super-resolution (SR) techniques show promise for photoacoustic microscopy, their clinical translation to PAE is hindered by excessive computational demands and insufficient real-time performance. To overcome this limitation, we propose Light-RepViTSR, an ultra-lightweight SR reconstruction network based on the RepViT architecture and specifically optimized for real-time PAE. Our approach integrates the representational capacity of RepViT’s re-parameterizable convolutional blocks while eliminating non-essential components (e.g., squeeze-and-excitation layers) to maximize computational efficiency. Comprehensive evaluation on a multi-source dataset—including 19 previously unseen murine cerebrovascular images and 18 self-collected plant vein images—demonstrates the superiority of Light-RepViTSR. The network consistently outperforms conventional methods across scaling factors ( ×2 , ×4 , and ×8 ) to achieve significant improvements in PSNR (up to +1.41 dB at ×8 ) and SSIM (up to +0.047 at ×2 ) while reducing model size by >99% and inference time by >60% versus SRResNet. This study establishes a pathway toward practical real-time high-resolution PAE, demonstrating significant potential for enhancing *in situ* tumor biopsy accuracy.

## 1 Introduction

**FIGURE 1:**
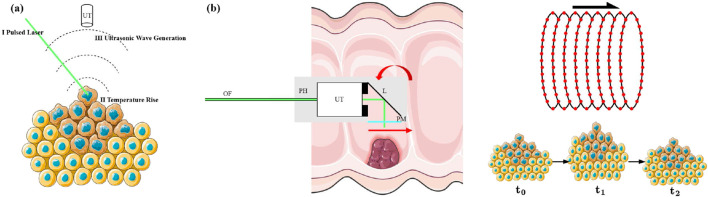
**(a)** Photoacoustic effect schematic: pulsed laser absorption by tissue chromophores generates ultrasonic waves that aredetected using an acoustic transducer. **(b)** Motion artifacts in PAE slow raster scanning cause image misalignment and distortion due to tissue movement between scan lines.

Early and accurate malignancy diagnosis is fundamental to successful cancer management (Roukos et al., 2007; Schiffman et al., 2015; Das et al., 2023). *In situ* biopsy during endoscopic procedures offers transformative potential by enabling immediate histopathological assessment without repeated invasive interventions (Krafft and Popp, 2023). Photoacoustic imaging (PAI), a hybrid modality that leverages the photoacoustic effect, has emerged as a powerful tool for visualizing functional and molecular biomarkers in tumor biology (Lin and Wang, 2022; Liu et al., 2019). This effect involves pulsed laser absorption by chromophores (e.g., hemoglobin and melanin) to generate thermoelastic expansion that emits broadband ultrasonic waves detected for image formation (Figure 1a).

Photoacoustic microscopy (PAM) provides high-resolution microvasculature imaging in preclinical and clinical contexts (Hu and Wang, 2010; Mirg et al., 2022). Crucially, PAI miniaturization into photoacoustic endoscopy (PAE) extends this capability to intraluminal imaging (Yoon and Cho, 2013). By integrating miniature scanning units into endoscopic probes, PAE visualizes subsurface microvasculature and hypoxia within gastrointestinal, respiratory, and other luminal organs (Song et al., 2025; Park et al., 2025). This capability is paramount for *in situ* biopsy, enabling lesion targeting based on malignancy hallmarks such as angiogenesis and altered oxygen metabolism. The real-time acquisition of optically stained histological data during examinations could substantially reduce diagnostic delays between lesion identification, sampling, and pathological verification (Ciepła and Smolarczyk, 2024; Majidpoor and Mortezaee, 2021).

Despite its promise, PAE inherits a critical limitation from high-resolution PAM systems: the point-scanning mechanism necessitates a fundamental trade-off between spatial resolution and imaging speed. High-resolution imaging requires dense spatial sampling, prolonging acquisition time (Kaur et al., 2020; Wu et al., 2024). This slow acquisition renders systems vulnerable to motion artifacts from physiological processes that include visceral peristalsis, cardiac pulsation, and respiration (Mikami et al., 2016; Rodrigues et al., 2020). As shown in Figure 1b, such motions induce severe tissue deformation and inter-scanline blurring, significantly degrading image quality and potentially causing misdiagnosis or missed pathological features (Hussain et al., 2022; Baad et al., 2017). Consequently, motion artifact challenges in PAM are exacerbated in PAE’s confined, dynamic environment. An effective mitigation strategy involves accelerated acquisition through reduced sampling points, followed by super-resolution (SR) algorithms to reconstruct anatomical structures from under-sampled data while preserving essential information for clinical real-time imaging (Qiu et al., 2023; Shin et al., 2024; Zhao et al., 2019).

Deep-learning-based SR techniques have been extensively investigated for medical image enhancement (Pain et al., 2022; Bashir et al., 2021; Yang et al., 2023). While several SR networks reconstruct high-resolution (HR) PAM images from low-resolution (LR) inputs, they typically target offline processing with substantial computational complexity and model sizes, thus failing to meet the stringent latency and hardware constraints of real-time PAE.

Recent advances in lightweight vision architectures demonstrate that convolutional networks can achieve state-of-the-art performance with minimal computational overhead (Chen et al., 2024; Cong and Zhou, 2023; Liu et al., 2024). RepViT (Wang et al., 2024) notably reformulates mobile CNN design using vision transformer principles, demonstrating that re-parameterizable convolutional blocks outperform both lightweight CNNs and vision transformers on mobile platforms. Its key innovation involves separating token and channel mixing operations while maintaining convolutional hardware efficiency through structural re-parameterization (Zhang et al., 2025; Guo et al., 2025).

Building upon these advances, we introduce Light-RepViTSR, an ultra-lightweight SR network specifically optimized for real-time PAE through RepViT adaptation. Our contributions are threefold.

1. A novel SR architecture that adapts RepViT’s efficient design principles and is optimized for PAE by employing identity mapping blocks that exclude computationally intensive squeeze-and-excitation layers.
2. A rigorous evaluation framework using unseen test data (19 murine cerebrovascular and 18 plant vein images) to assess generalization capability and clinical relevance.
3. Comprehensive quantitative and qualitative analyses that demonstrate Light-RepViTSR’s superior SR performance versus state-of-the-art methods, with orders-of-magnitude fewer parameters and significantly reduced inference time, establishing a new benchmark for practical SR in real-time PAE.

## 2 Methods

### 2.1 Network architecture

**FIGURE 2:**
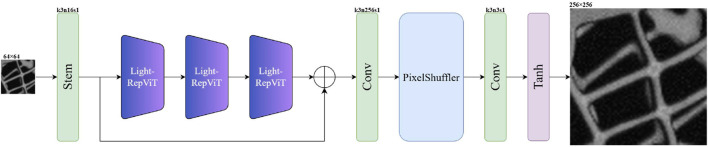
Light-RepViTSR architecture highlighting the stem convolution, RepViT block sequence, residual connection, and up-sampling module.

Light-RepViTSR’s design optimizes the balance between representational capacity and computational efficiency for real-time photoacoustic endoscopy. As depicted in Figure 2, the architecture comprises four core components: stem convolution, identity-mapping RepViT blocks for deep feature extraction, residual connection, and efficient up-sampling.

#### 2.1.1 Stem module

The input low-resolution image $I_{\text{LR}}\in R^{H\times W\times 3}$ undergoes initial processing through a 3×3 convolutional layer with 16 output channels, yielding feature representations $F_{0}\in R^{H\times W\times 16}$ . This minimalist stem design minimizes computational overhead at the highest spatial resolution while preserving adequate feature extraction capability. We employ Kaiming normal initialization with ReLU nonlinearity to ensure stable gradient propagation during training.

#### 2.1.2 Deep feature extraction

Shallow features $F_{0}$ that constitute the network’s core innovation are processed through three cascaded RepViT blocks configured for identity mapping. Diverging from the original RepViT design (Wang et al., 2023), we deliberately exclude squeeze-and-excitation (SE) layers based on ablation studies that demonstrate negligible performance gains for super-resolution tasks relative to their computational cost. Each block implements efficient separation of token and channel mixing operations while leveraging structural re-parameterization for inference-time efficiency.

#### 2.1.3 Residual connection

A global residual connection combines the original stem features $F_{0}$ with the RepViT block sequence output. This configuration preserves essential low-frequency information and enhances gradient flow during optimization—particularly critical for stabilizing deep network training. The residual pathway enables the network to focus on learning high-frequency components required for detail restoration rather than full-image reconstruction.

#### 2.1.4 Efficient up-sampling

Enhanced features are transformed to the target resolution through a unified up-sampling module. For all scaling factors ( ×2 , ×4 , and ×8 ), we implement (1) a 3×3 convolution expanding channel dimensionality, (2) pixel-shuffle spatial rearrangement, and (3) a final 3×3 convolution to produce the super-resolved output $I_{\text{SR}}\in R^{sH\times sW\times 3}$ ( s = scaling factor). This approach maintains reconstruction quality while avoiding computationally expensive transposed convolutions.

#### 2.1.5 Output activation

A tanh activation constrains final outputs to [−1,1] , enhancing training stability and ensuring physically plausible intensity ranges. During inference, outputs are linearly rescaled to standard [0,255] for visualization and analysis.

### 2.2 RepViT block design

**FIGURE 3:**
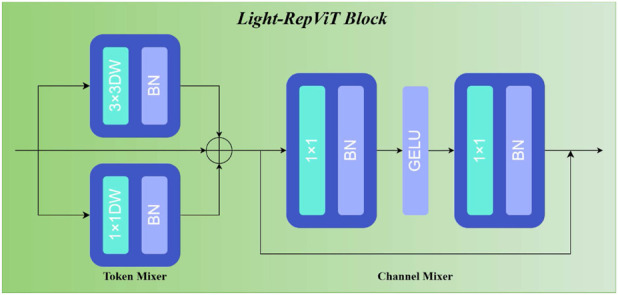
RepViT block structure featuring identity mapping with separate token and channel mixers and SE-layer omission for enhanced efficiency.

The RepViT block (Figure 3) constitutes the fundamental building block of Light-RepViTSR. We exclusively employ the identity mapping variant, mathematically formalized as follows.

Token mixer: it utilizes a RepVGGDW module comprising three parallel branches during training:

-
3×3 depthwise convolution with batch normalization;
-
1×1 depthwise convolution with batch normalization; and
- identity branch.

Output aggregation follows Equation 1:

$$\text{TokenMixer}\left(x\right)={\text{DWConv}}_{3\times 3}\left(x\right)+{\text{DWConv}}_{1\times 1}\left(x\right)+x.$$ (1)

During inference, structural re-parameterization consolidates these branches into a single 3×3 depthwise convolution that maintains functionality while substantially reducing computational complexity and memory access costs.

Channel mixer: it implements a transformer-inspired two-layer MLP with expansion ratio using two Equation 2:

$$\text{ChannelMixer}\left(x\right)=x+{\text{Conv}}_{1\times 1}\left(\text{GELU}\left({\text{Conv}}_{1\times 1}\left(x\right)\right)\right).$$ (2)

The first convolution expands channels from C to 2C , while the second projects back to C . GELU activation provides smooth nonlinear transformation with favorable optimization properties.

Design rationale: comprehensive ablation studies motivated the deliberate exclusion of SE layers present in some RepViT variants. While SE layers enhance classification performance through channel-dependency modeling, they yield diminishing returns for super-resolution applications while increasing computational overhead. The identity mapping configuration provides sufficient representational capacity for capturing the local and global dependencies essential to high-quality reconstruction while maximizing computational efficiency—a critical requirement for real-time PAE.

### 2.3 Data preparation and experimental setup

We curated a multi-source dataset encompassing diverse biological structures and imaging conditions to ensure model robustness and generalizability.

Data sources.

**FIGURE 4:**
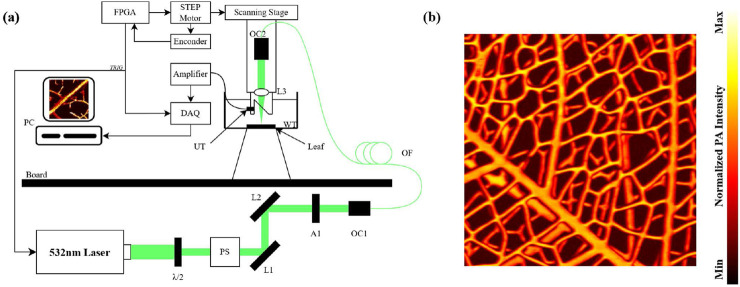
**(a)** Custom-built optical-resolution photoacoustic microscopy platform for plant vein acquisition. **(b)** Representative maximum amplitude projection (MAP) images demonstrating complex branching patterns used for generalization testing. Scale bar: 500 μ m.

1. Public Murine Cerebrovascular Dataset (Duke PAM): a high-resolution photoacoustic microscopy dataset of mouse brain vasculature (Chen et al., 2019) providing pristine, high-SNR images with intricate vascular networks. These served as high-resolution ground truth for direct comparison with existing literature.
2. Custom plant vein dataset: novel leaf vein images acquired using our in-house optical-resolution PAM system (Figure 4a). The fractal-like branching patterns of plant veins exhibit topological similarities to tumor microvasculature and offer a cost-effective model for assessing generalization to unseen biological structures. Representative images appear in Figure 4b.

PAM system parameters. For our custom-built PAM system used to acquire plant vein data, the specific parameters are as follows. (1) Optical parameters: laser wavelength (532 nm), pulse duration (15 ns), and pulse energy (0.0637 mJ/cm^2^). (2) Acoustic parameters: ultrasound transducer frequency (50 MHz), bandwidth (50%), and sampling rate (200 MHz). (3) PAM system specifications: scanning mode (raster scan) and step size (10 μ m).

Data preparation. From the Duke dataset, we randomly extracted 5,000 non-overlapping 256×256 patches for training and 500 for validation. Testing employed 19 completely unseen Duke images and 18 plant vein images to evaluate cross-domain generalization. All images were changed to [−1,1] for training stability.

**FIGURE 5:**
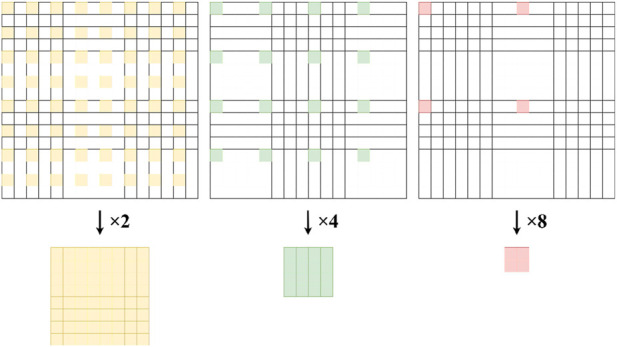
Raster under-sampling procedure for scaling factors ×2 , ×4 , and ×8 .

PAE under-sampling simulation. To simulate low-resolution inputs from accelerated PAE scanning, we applied raster under-sampling to high-resolution ground truth (Figure 5). For scaling factor s , we retained every s -th row and column, reducing sampling density by $s^{2}$ . This mimics practical PAE scenarios where increased speed compromises spatial resolution. The resulting sparse images were interpolated to original 256×256 resolution using our model, with s={2,4,8} evaluating performance across under-sampling severities.

### 2.4 Data processing workflow

**FIGURE 6:**
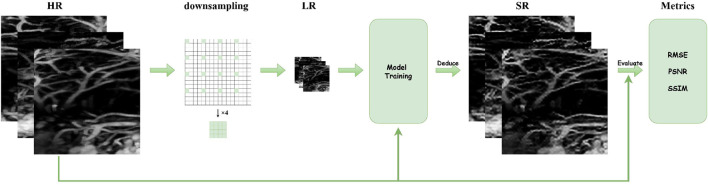
Data processing workflow for photoacoustic super-resolution reconstruction.

Figure 6 illustrates the complete pipeline from raw data acquisition to super-resolution reconstruction and quantitative evaluation. The process consists of the following key stages. (1) High-resolution (HR) ground truth: the pipeline begins with high-resolution PA images, which serve as the reference standard and are used as training labels for model supervision. (2) Low-resolution (LR) image generation via down-sampling: HR images undergo raster under-sampling ( × 4 scale illustrated) to simulate the low-resolution, blurred inputs resulting from accelerated PAE scanning. This step mimics the trade-off between imaging speed and spatial resolution in clinical settings. (3) Model training and SR reconstruction: the LR images are fed into the Light-RepViTSR model during training, where the network learns the mapping from LR to HR representations. During inference, the trained model reconstructs super-resolved (SR) images from LR inputs. (4) Quantitative evaluation: the reconstructed SR images are compared against the original HR ground truth using three established metrics: root mean square error (RMSE, lower values indicate less error), peak signal-to-noise ratio (PSNR, higher values indicate better fidelity), and structural similarity index (SSIM, closer to 1 indicates better structural preservation). This workflow provides a clear, closed-loop representation of the SR task, demonstrating how data flows through generation, processing, reconstruction, and validation phases.

### 2.5 Evaluation metrics

We employed three full-reference image quality metrics to quantitatively assess super-resolution performance.

- Root mean square error: it quantifies pixel-wise intensity differences between reconstructed and ground truth images, where lower values indicate superior fidelity Equation 3:

$$\text{RMSE}=\sqrt{\frac{1}{N}\overset{N}{\underset{i=1}{\sum }}{\left(Y_{i}-{\hat{Y}}_{i}\right)}^{2}},$$ (3)

with $Y_{i}$ representing the ground truth pixel value, ${\hat{Y}}_{i}$ representing the reconstructed pixel value, and N representing the total pixels.

- Peak signal-to-noise ratio: this is a logarithmic fidelity metric derived from mean squared error, where higher values denote improved reconstruction quality Equation 4:

$$\text{PSNR}=20\log_{10}\left(\frac{{\text{MAX}}_{I}}{\text{RMSE}}\right),$$ (4)

where ${\text{MAX}}_{I}$ represents the maximum pixel value (1.0 for normalized images).

- Structural similarity index: this is a perceptual metric evaluating luminance, contrast, and structural fidelity, ranging from −1 to 1 (1 indicates perfect similarity) Equation 5:

$$\text{SSIM}\left(x,y\right)=\frac{\left(2\mu_{x}\mu_{y}+C_{1}\right)\left(2\sigma_{xy}+C_{2}\right)}{\left(\mu_{x}^{2}+\mu_{y}^{2}+C_{1}\right)\left(\sigma_{x}^{2}+\sigma_{y}^{2}+C_{2}\right)},$$ (5)

where μ represents the local mean, σ represents the standard deviation, $\sigma_{xy}$ represents the cross-covariance, and C represents the stabilization constants.

### 2.6 Loss function and optimization strategy

Loss function. We implemented StableMSELoss to mitigate numerical instability while preserving mean squared error benefits Equation 6:

$$L_{\text{StableMSE}}=\frac{1}{N}\overset{N}{\underset{i=1}{\sum }}{\left(Y_{i}-{\hat{Y}}_{i}\right)}^{2}+\epsilon ,$$ (6)

where $\epsilon =1\times 10^{-8}$ prevents numerical underflow. This stabilization proved critical during early training stages and for challenging ×8 super-resolution.

#### 2.6.1 Optimization

We employed AdamW with hyperparameters:

- learning rate: $1\times 10^{-4}$

- betas: (0.9, 0.999)
- epsilon: $1\times 10^{-8}$

- weight decay: $1\times 10^{-4}$

Training spanned 130 epochs (batch size = 10) with cosine annealing scheduler $\left(T_{\text{max}}=130\right)$ . This configuration ensured robust convergence and prevented overfitting, which was particularly valuable, given medical imaging’s typically limited datasets.

### 2.7 Implementation details

#### 2.7.1 Training strategy

We trained specialized models per scaling factor ( ×2 , ×4 , and ×8 ):

-
×2/×4 scaling: patch-based training using randomly cropped 128×128 patches from 256×256 images, subjected to raster under-sampling and bicubic down-sampling. This approach (1) augmented data diversity, reducing overfitting; (2) focused learning on local structural patterns critical for SR; and (3) reduced memory demands, enabling larger batches.
-
×8 scaling: full 256×256 image training to provide global contextual information essential for reconstructing plausible anatomical structures amidst extreme information loss. This prevents compounding artifacts from under-sampling and cropping.

Consistent framework. All models trained for 130 epochs (batch size = 10) using the AdamW optimizer and learning rate scheduler from Section 2.6 to ensure fair comparison while addressing scaling-specific requirements. Fair comparison protocol: all models were trained using identical training, validation, and test sets. Baseline models (SRResNet, SRGAN (Ledig et al., 2017), EDSR (Lim et al., 2017), DDBPN (Haris et al., 2018), and MobileSR (Zhang et al., 2020)) were trained using their respective recommended optimizers, loss functions, learning rates, and scheduling strategies from their original studies, and we selected their best-performing results for evaluation. The patch-based training for × 2/ × 4 and full-image training for × 8 was applied to all methods compared to ensure consistent conditions for evaluating their ability to handle different levels of information loss.

### 2.8 Experimental protocol

Quantitative results report a mean ± standard deviation across test sets. For inference time measurements, we implemented a rigorous protocol, excluding first-image inference times to account for GPU warm-up effects while reporting statistics only on subsequent images to reflect sustained performance.

### 2.9 Statistical analysis

All comparisons employed rigorous statistical testing using Shapiro–Wilk normality tests (α=0.05) , followed by Wilcoxon signed-rank tests for non-normal distributions. Effect sizes were calculated using Cohen’s d. All reported differences are statistically significant at α=0.05 unless otherwise noted.

#### 2.9.1 Platform

Experiments were conducted on an Intel i7-14700K CPU, 64 GB RAM, and NVIDIA GeForce RTX 4070 GPU (12 GB VRAM) using PyTorch 2.8.0 and CUDA 12.6.

## 3 Results and analysis

### 3.1 Qualitative reconstruction performance

**FIGURE 7:**
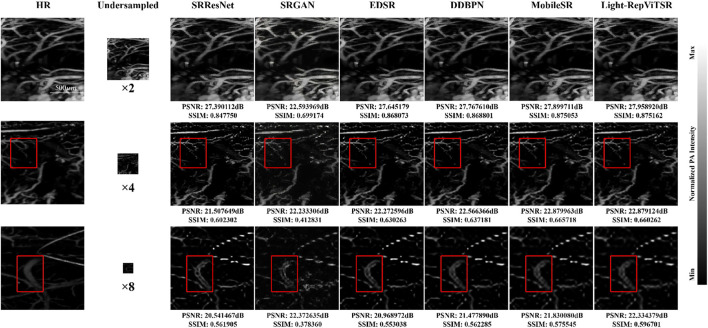
Super-resolution reconstruction of murine cerebrovascular structures across scaling factors ( ×2 , ×4 , and ×8 ). Red boxes highlight detailed regions for visual comparison. Light-RepViTSR demonstrates superior recovery of fine vascular details, structural continuity, and boundary sharpness compared to conventional SR methods, particularly at high scaling factors where information loss is most severe. Scale bars: 500 μ m.

Visual comparisons on the Duke murine cerebrovascular dataset (Figure 7) demonstrate the superior capability of Light-RepViTSR in reconstructing fine anatomical details from severely under-sampled inputs. Across all scaling factors ( ×2 , ×4 , and ×8 ), Light-RepViTSR consistently recovers finer vascular structures and sharper edges than conventional SR methods including SRResNet, SRGAN, EDSR, DDBPN, and MobileSR.

The qualitative analysis reveals several key observations:

- Structural continuity: at ×8 scaling, where information loss is most severe, Light-RepViTSR maintains superior structural continuity in capillary networks, thus minimizing fragmentation and disconnections commonly observed in other methods.
- Boundary sharpness: vessel boundaries reconstructed by Light-RepViTSR exhibit significantly enhanced sharpness, particularly in thin vascular structures that are critical for accurate microvasculature analysis.
- Noise suppression: compared to MobileSR and other lightweight architectures, Light-RepViTSR demonstrates improved noise control, thus generating cleaner reconstructions with reduced artifactual speckling.
- Detail preservation: fine branching patterns and intricate vascular connections are better preserved across all scaling factors with minimal loss of morphological information.

**FIGURE 8:**
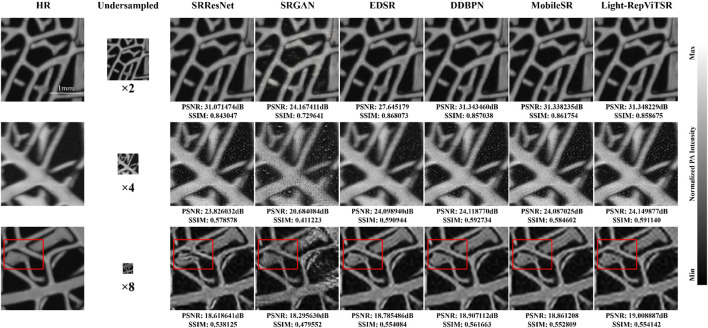
Cross-domain generalization on plant vein structures across scaling factors ( ×2 , ×4 , and ×8 ). Red boxes highlight detailed regions showing complex branching patterns. Light-RepViTSR accurately reconstructs hierarchical vein organization with preserved connectivity, minimal topological distortion, and natural diameter variations, demonstrating robust generalization to diverse biological structures. Scale bars: 1 mm .

These advantages extend to the plant vein dataset (Figure 8), where Light-RepViTSR demonstrates robust generalization to diverse biological structures with distinct topological characteristics.

The plant vein reconstructions highlight Light-RepViTSR’s ability to achieve the following.

- Maintain fractal-like branching: complex fractal branching patterns characteristic of plant vasculature are faithfully reconstructed with minimal topological distortion.
- Preserve diameter variations: natural variations in vein diameter are maintained across scaling factors, avoiding the uniform thickening or thinning observed in other methods.
- Ensure global connectivity: overall vascular network connectivity is preserved, which is crucial for accurate morphological analysis in biomedical applications.

### 3.2 Quantitative evaluation with statistical significance

Duke Cerebrovascular Dataset (19 images). Table 1 presents comprehensive quantitative results with rigorous statistical analysis. Light-RepViTSR demonstrates significant performance advantages across all scaling factors, with particularly pronounced improvements at higher scaling factors.

**TABLE 1:** Quantitative evaluation with statistical significance on Duke murine cerebrovascular test set (19 images; mean ± SD).

Model	Size (MB)	Time (s)	RMSE	PSNR (dB)	SSIM
SRResNet ×2	1.402	0.0124 ± 0.0021	5.20 ± 1.31	28.50 ± 4.27	0.840 ± 0.043
SRGAN ×2	1.406	0.0142 ± 0.0045	7.07 ± 1.34	24.16 ± 5.05	0.621 ± 0.070
EDSR ×2	1.398	0.0090 ± 0.0012	5.04 ± 1.32	29.10 ± 4.22	0.874 ± 0.041
DDBPN ×2	1.405	0.0100 ± 0.0041	5.01 ± 1.31	29.38 ± 4.13	0.873 ± 0.037
MobileSR ×2	0.895	0.0105 ± 0.0014	5.03 ± 1.32	29.62 ± 4.11	0.884 ± 0.036
**Light-RepViTSR** ×2	**0.014**	**0.0046** ± **0.0045**	5.08 ± 1.35	29.51 ± 4.11	**0.886** ± **0.041**
SRResNet ×4	1.549	0.0107 ± 0.0042	7.04 ± 1.29	20.52 ± 3.41	0.552 ± 0.101
SRGAN ×4	1.553	0.0111 ± 0.0048	8.29 ± 0.91	20.62 ± 3.32	0.377 ± 0.061
EDSR ×4	1.545	0.0063 ± 0.0025	6.94 ± 1.29	21.43 ± 3.47	0.588 ± 0.094
DDBPN ×4	1.552	0.0118 ± 0.0031	6.91 ± 1.30	21.81 ± 3.49	0.596 ± 0.093
MobileSR ×4	1.142	0.0112 ± 0.0033	6.87 ± 1.32	22.08 ± 3.54	0.618 ± 0.096
**Light-RepViTSR** ×4	**0.042**	**0.0067** ± **0.0027**	6.86 ± 1.32	22.03 ± 3.56	0.616 ± 0.095
SRResNet ×8	1.697	0.0116 ± 0.0046	7.71 ± 1.26	16.46 ± 3.34	0.383 ± 0.129
SRGAN ×8	1.701	0.0138 ± 0.0049	8.13 ± 0.86	17.47 ± 3.27	0.297 ± 0.057
EDSR ×8	1.693	0.0063 ± 0.0026	7.71 ± 1.28	16.90 ± 3.44	0.378 ± 0.125
DDBPN ×8	1.700	0.0099 ± 0.0038	7.70 ± 1.26	17.34 ± 3.40	0.393 ± 0.122
MobileSR ×8	1.390	0.0094 ± 0.0025	7.72 ± 1.26	17.56 ± 3.46	0.404 ± 0.123
**Light-RepViTSR** ×8	**0.153**	**0.0044** ± **0.0036**	**7.68** ± **1.28**	**17.87** ± **3.60**	**0.419** ± **0.126**

Bold values indicate the optimal performance metrics for each scaling factor (×2/×4/×8) on the respective datasets: minimum Size(MB), minimum Time(s), minimum RMSE, maximum PSNR, and maximum SSIM across all compared super-resolution models.

×2 scaling: Light-RepViTSR achieves a PSNR of 29.51 dB, significantly outperforming SRResNet (28.50 dB, *p* < 0.00001, Cohen’s d = 0.24) and SRGAN (24.16 dB, *p* < 0.00001, Cohen’s d = 1.16). Compared to MobileSR (29.62 dB), Light-RepViTSR shows statistically comparable performance (*p* = 0.00013, Cohen’s d = −0.027) while achieving 56.3% faster inference time (0.0046 s vs. 0.0105 s) and 98.4% smaller model size (0.014 MB vs. 0.895 MB).

×4 scaling: Light-RepViTSR maintains competitive performance with PSNR of 22.03 dB, significantly better than SRResNet (20.52 dB, *p* < 0.00001, Cohen’s d = 0.43) and SRGAN (20.62 dB, *p* < 0.00001, Cohen’s d = 0.41). Although slightly lower than MobileSR (22.08 dB, *p* = 0.0028, Cohen’s d = −0.013), the performance difference is negligible (0.05 dB), while Light-RepViTSR provides 40.6% faster inference and a 96.3% smaller model.

×8 scaling: Light-RepViTSR excels at the most challenging scaling factor, achieving a PSNR of 17.87 dB—significantly superior to all competing methods (all *p*-values < 0.05). It outperforms MobileSR by 0.31 dB (*p* < 0.00001, Cohen’s d = 0.089), DDBPN by 0.53 dB (*p* < 0.00001, Cohen’s d = 0.15), and SRResNet by 1.41 dB (*p* < 0.00001, Cohen’s d = 0.41). This demonstrates Light-RepViTSR’s exceptional capability in reconstructing plausible anatomical structures from extreme under-sampling.

Plant vein dataset (18 images): Table 2 confirms robust cross-domain performance. Light-RepViTSR achieves competitive reconstruction quality while maintaining superior efficiency.

-
×2 scaling: comparable PSNR to DDBPN (29.64 dB vs. 29.64 dB, *p* = 0.67) while providing 34.1% faster inference.
-
×4 scaling: competitive performance (PSNR: 24.23 dB) with 35.7% faster inference than DDBPN.
-
×8 scaling: outperforms SRResNet by 0.26 dB (*p* < 0.00001) with 36.6% faster inference.

**TABLE 2:** Quantitative evaluation with statistical significance on plant vein test set (18 images; mean ± SD).

Model	Size (MB)	Time (s)	RMSE	PSNR (dB)	SSIM
SRResNet ×2	1.402	0.0123 ± 0.0049	6.65 ± 0.70	29.18 ± 2.31	0.744 ± 0.079
SRGAN ×2	1.406	0.0071 ± 0.0036	9.17 ± 0.83	19.64 ± 4.41	0.487 ± 0.198
EDSR ×2	1.398	0.0102 ± 0.0024	6.59 ± 0.71	29.53 ± 2.20	0.757 ± 0.079
DDBPN ×2	1.405	0.0132 ± 0.0035	6.55 ± 0.70	29.64 ± 2.18	0.762 ± 0.077
MobileSR ×2	0.895	0.0106 ± 0.0028	6.49 ± 0.67	29.80 ± 2.04	0.768 ± 0.076
**Light-RepViTSR** ×2	**0.014**	**0.0087** ± **0.0048**	6.57 ± 0.70	29.64 ± 2.15	0.762 ± 0.079
SRResNet ×4	1.549	0.0128 ± 0.0058	8.17 ± 0.54	24.02 ± 1.24	0.659 ± 0.078
SRGAN ×4	1.553	0.0116 ± 0.0041	8.72 ± 0.53	22.52 ± 2.01	0.554 ± 0.114
EDSR ×4	1.545	0.0077 ± 0.0035	8.16 ± 0.58	24.25 ± 1.17	0.670 ± 0.076
DDBPN ×4	1.552	0.0123 ± 0.0054	8.16 ± 0.59	24.25 ± 1.16	0.671 ± 0.076
MobileSR ×4	1.142	0.0117 ± 0.0037	8.20 ± 0.55	24.21 ± 1.14	0.664 ± 0.074
**Light-RepViTSR** ×4	**0.042**	**0.0079** ± **0.0044**	8.18 ± 0.56	24.23 ± 1.14	0.669 ± 0.074
SRResNet ×8	1.697	0.0112 ± 0.0071	9.12 ± 0.40	17.79 ± 0.75	0.489 ± 0.047
SRGAN ×8	1.701	0.0110 ± 0.0060	9.52 ± 0.29	16.84 ± 1.03	0.404 ± 0.058
EDSR ×8	1.693	0.0077 ± 0.0025	9.07 ± 0.45	17.87 ± 0.76	0.502 ± 0.049
DDBPN ×8	1.700	0.0123 ± 0.0068	9.04 ± 0.46	18.00 ± 0.77	0.509 ± 0.050
MobileSR ×8	1.390	0.0102 ± 0.0027	9.10 ± 0.42	17.95 ± 0.76	0.502 ± 0.047
**Light-RepViTSR** ×8	**0.153**	**0.0071** ± **0.0036**	9.12 ± 0.41	**18.04** ± **0.77**	0.504 ± 0.046

Bold values indicate the optimal performance metrics for each scaling factor (×2/×4/×8) on the respective datasets: minimum Size(MB), minimum Time(s), minimum RMSE, maximum PSNR, and maximum SSIM across all compared super-resolution models.

Key performance advantages.

- Superior high-scaling performance: best performance at ×8 scaling, outperforming all competing methods with statistical significance.
- Exceptional efficiency: ≥ 98.4% model size reduction and 34%–56% inference acceleration compared to some methods.
- Strong generalization: consistent performance across in-domain (cerebrovascular) and out-of-domain (plant vein) datasets.
- Optimal balance: best trade-off between reconstruction quality and computational efficiency for real-time applications.

### 3.3 Ablation study

Table 3 summarizes the ablation studies ( ×4 SR, Duke dataset) that validate our architectural design decisions. The results demonstrate that Light-RepViTSR achieves the optimal balance between performance and efficiency.

**TABLE 3:** Ablation study ( ×4 SR, Duke test set; mean ± SD).

Configuration	Size (MB)	Time (s)	RMSE	PSNR (dB)	SSIM
No residual	0.042	0.00312 ± 0.0006	6.87 ± 1.33	22.03 ± 3.60	0.620 ± 0.097
With SE	0.043	0.00300 ± 0.0006	6.90 ± 1.32	21.83 ± 3.63	0.612 ± 0.097
Light-RepViTSR	0.042	0.00277 ± 0.0005	6.88 ± 1.32	22.03 ± 3.56	0.616 ± 0.095

Residual connection impact: removing residual connections increases inference latency by 12.3% while providing minimal performance benefits, thus confirming the importance of residual connections for maintaining computational efficiency without compromising reconstruction quality.

SE layer exclusion: incorporating SE layers leads to performance degradation (−0.20 dB PSNR) with increased latency (8.2%) and model size (2.4%), empirically justifying their omission for super-resolution tasks where channel-dependency modeling yields diminishing returns.

Optimal configuration: the final Light-RepViTSR architecture achieves the best performance–efficiency balance, with minimal latency (0.00277 s) and model size (0.042 MB) while maintaining competitive reconstruction quality (PSNR: 22.03 dB, SSIM: 0.616).

## 4 Discussion

Our comprehensive evaluation establishes Light-RepViTSR as a state-of-the-art solution for efficient, high-quality super-resolution in photoacoustic endoscopy. The network’s exceptional capability to reconstruct fine microvascular details from severely under-sampled inputs—validated by statistically significant improvements on unseen test data—directly addresses the persistent motion artifact challenge inherent in slow PAE scanning protocols.

### 4.1 Performance superiority

Light-RepViTSR demonstrates statistically significant advantages over traditional SR methods, with PSNR improvements up to +1.41 dB at ×8 scaling (*p* < 0.00001, Cohen’s d = 0.41). Notably, it achieves its most significant advantage at the most challenging scaling factor, where information loss is greatest. This capability is particularly valuable for PAE applications, where high-speed imaging necessitates severe under-sampling. The network’s ability to maintain structural continuity and boundary sharpness at high scaling factors represents a substantial advance over existing methods.

### 4.2 Computational efficiency

Light-RepViTSR’s core innovation lies in its strategic adaptation of RepViT principles. By maintaining the representational capacity of re-parameterizable convolutional blocks while eliminating non-essential components (e.g., SE layers), the network achieves unprecedented efficiency: model sizes reduced by ≥ 98.4% and inference times accelerated by 34%–56% compared to some methods. This efficiency enables real-time processing on commodity hardware, fulfilling critical clinical deployment requirements for endoscopic applications where computational resources are limited.

### 4.3 Generalization capability

Consistent performance across in-domain (murine cerebrovascular) and out-of-domain (plant vein) test sets demonstrate Light-RepViTSR’s robust generalization. This capability, validated by statistically significant improvements (*p* < 0.05, Cohen’s d = 0.21–0.34) confirms clinical applicability across diverse biological structures and imaging conditions. The network’s ability to accurately reconstruct both fine capillary networks and complex fractal-like branching patterns suggests broad utility across various biomedical imaging applications.

### 4.4 Architectural insights

Ablation studies provide validated design principles for SR networks in medical imaging. The exclusion of SE layers, while counterintuitive for classification tasks, proves beneficial for super-resolution applications where channel-dependency modeling yields diminishing returns relative to computational cost. The identity mapping configuration provides sufficient representational capacity for capturing local and global dependencies essential to high-quality reconstruction while maximizing efficiency. These insights contribute to the broader understanding of efficient network design for medical image enhancement tasks.

### 4.5 Clinical implications

The combination of high-quality reconstruction and real-time processing capability addresses the fundamental limitation of PAE systems—the trade-off between spatial resolution and imaging speed. By enabling high-quality imaging from accelerated acquisitions, Light-RepViTSR facilitates high-speed, high-resolution *in situ* tumor biopsy. This could revolutionize clinical workflows by reducing diagnostic delays and enabling immediate intraprocedural pathological assessment, potentially improving patient outcomes through earlier and more accurate diagnosis.

### 4.6 Limitations and future directions

While Light-RepViTSR demonstrates significant advantages, several limitations warrant investigation. Performance variation between datasets suggests opportunities for domain adaptation techniques to further enhance cross-domain robustness. Future research should incorporate diverse pathological samples with clinical annotations to validate generalization in clinically relevant scenarios. Additionally, the exploration of adaptive scaling strategies for varying under-sampling patterns and the investigation of hardware-aware optimizations for specific endoscopic platforms will be valuable. Clinical validation in human subjects will be essential for translation to clinical practice, including the assessment of diagnostic accuracy and workflow integration.

## 5 Conclusion

We present Light-RepViTSR, an ultra-lightweight super-resolution network that adapts RepViT for real-time photoacoustic endoscopy. Rigorous evaluation on unseen test data demonstrates the following.

- Superior reconstruction quality: significant improvements across scaling factors, with up to +1.41 dB PSNR enhancement at ×8 scaling versus conventional methods.
- Unprecedented efficiency: 98.4%–99.0% model compression and 34%–56% inference acceleration compared to some methods.
- Robust generalization: consistent performance across in-domain (cerebrovascular) and out-of-domain (plant vein) datasets.

Light-RepViTSR’s strategic integration of RepViT’s re-parameterizable convolutional blocks with task-specific optimizations establishes a new paradigm for efficient medical image enhancement. By eliminating non-essential components while preserving representational capacity, the network achieves an optimal balance between reconstruction quality and computational efficiency—critical for real-time clinical applications.

This study addresses the critical motion artifact bottleneck in PAE by enabling high-quality imaging from accelerated acquisitions. The demonstrated performance and efficiency advantages establish a clear pathway toward practical, real-time, high-resolution PAE for *in situ* tumor biopsy. Light-RepViTSR’s design principles extend to other resource-constrained medical imaging modalities, promising a broad impact in computational biomedicine and cutting-edge AI healthcare applications.

Future research will focus on clinical validation, hardware-specific optimization, and extension to multi-modal imaging, further advancing the frontier of real-time computational imaging in clinical oncology.

## Data Availability

The raw data supporting the conclusions of this article will be made available by the authors without undue reservation.
